# Exogenous Methyl Jasmonate Improves Heat Tolerance of Perennial Ryegrass Through Alteration of Osmotic Adjustment, Antioxidant Defense, and Expression of Jasmonic Acid-Responsive Genes

**DOI:** 10.3389/fpls.2021.664519

**Published:** 2021-05-07

**Authors:** Yanning Su, Yizhi Huang, Xintan Dong, Ruijia Wang, Mingyu Tang, Jiabang Cai, Jiayi Chen, Xinquan Zhang, Gang Nie

**Affiliations:** Department of Forage Science, College of Grassland Science and Technology, Sichuan Agricultural University, Chengdu, China

**Keywords:** perennial ryegrass, heat tolerance, methyl jasmonic acid, gene expression, growth regulator

## Abstract

Perennial ryegrass (*Lolium perenne* L.) is an important cool-season grass species that is widely cultivated in temperate regions worldwide but usually sensitive to heat stress. Jasmonates (JAs) may have a positive effect on plant tolerance under heat stress. In this study, results showed that exogenous methyl jasmonic acid (MeJA) could significantly improve heat tolerance of perennial ryegrass through alteration of osmotic adjustment, antioxidant defense, and the expression of JA-responsive genes. MeJA-induced heat tolerance was involved in the maintenance of better relative water content (RWC), the decline of chlorophyll (Chl) loss for photosynthetic maintenance, as well as maintained lower electrolyte leakage (EL) and malondialdehyde (MDA) content under heat condition, so as to avoid further damage to plants. Besides, results also indicated that exogenous MeJA treatment could increase the activities of superoxide dismutase (SOD), peroxidase (POD), catalase (CAT), and ascorbate peroxidase (APX), thus enhancing the scavenging ability of reactive oxygen species, alleviating the oxidative damage caused by heat stress. Heat stress and exogenous MeJA upregulated transcript levels of related genes (*LpLOX2*, *LpAOC*, *LpOPR3*, and *LpJMT*) in JA biosynthetic pathway, which also could enhance the accumulation of JA and MeJA content. Furthermore, some NAC transcription factors and heat shock proteins may play a positive role in enhancing resistance of perennial ryegrass with heat stress.

## Introduction

Perennial ryegrass (*Lolium perenne* L.) is native to Asia, Europe, and northern Africa and becomes a crucial grass species widely cultivated in temperate regions as high-quality forage and turfgrass (Yu et al., 2015). It has many desirable agronomic qualities, such as long growing season with strong adaptability, rapid establishment as a primary turf species, and high forage yield under suitable environments and conditions (Casler et al., 1976). However, it is reported that perennial ryegrass is generally sensitive to heat stress, so that it does not withstand hot weather, especially in warmer regions (Yu et al., 2013; Zhang et al., 2017). High temperature always affects the balance of growth and development by accelerating and redirecting metabolic processes (Wang et al., 2017; Li et al., 2020). Furthermore, the regions planting perennial ryegrass even experience high temperature over 38°C in summer, which greatly exceeds the temperature of these cool-season grasses for growth and reduces the forage yield and lawn quality (Wang et al., 2015).

Plant adaptation to adversity is controlled by both genetic and hormonal factors. To date, the use of plant hormones such as jasmonic acid (JA) to improve the growth of turfgrass and enhancing resistance is increasingly concerning in recent years. JA and its derivatives including methyl jasmonic acid (MeJA) together are called jasmonates (JAs). Many studies have shown that JA substances have a wide range of physiological effects on plant growth, development, and abiotic tolerance, such as inhibiting plant growth and pollen germination, promoting leaf senescence, and fruit maturation (Sembdner and Parthier, 1993; Creelman and Mullet, 1995; Arooran et al., 2019). Besides, as an endogenous signal molecule, it is involved in plant resilience to mechanical injury, pests and diseases, drought, high salinity, low temperature, and other conditions (Jiang et al., 2017). Previous study has shown that the increasing level of MeJA induces a JA-dependent defense response relating to the enhancement of secondary metabolism (Cheong and Yang, 2003). When plant is injured, the amount of MeJA increases significantly, which promotes the biosynthesis of some substances related to environmental stress (such as hormone and proline) and induces the expression of a series of genes related to stress tolerance, thus enhancing the plant resistance (Wasternack, 2014). JAs are derived from α -linolenic acid (α-LeA, 18:3), whose formation in plastids is catalyzed by fatty acid desaturase (*FAD*) and phospholipase A1 (*PLA*). Subsequently a-LeA is converted to cis-(+)-12-oxophytodienoic acid (*OPDA*) by enzymes including lipoxygenase (*LOX*), allene oxide synthase (*AOS*), allene oxide cyclase (*AOC*), and 12-oxo-phytodienoic acid reductase (*OPR*), then undergoes three rounds of β-oxidation to form JA (Turner et al., 2002). The expression of several key enzyme genes in the JA synthesis pathway has a great impact on JA level in plants. In addition, trauma and other stress factors that induce the JA response could also promote the expression of these genes, and the activation of these gene transcription occurs at the site of JA synthesis (Dhondt et al., 2000; Halitschke and Baldwin, 2003; Stenzel et al., 2003; Matsul, 2006; Tomoyuki et al., 2008).

*NAC* genes play various roles as transcription factors in multiple plants during growth and developmental processes, as well as diverse defense responses (Olsen et al., 2005). It has been reported that overexpression of *OsNAC10* enhanced rice (*Oryza sativa* L.) resistance to drought, high salinity, and low temperature significantly (Jeong et al., 2010; Sun et al., 2013) and also responded to salicylic acid and MeJA treatment (Zhou et al., 2013; Liang et al., 2014). For a cell to survive under heat stress, it is quite important to prevent upholding the proteins in their functional conformation and foreign proteins collection (Nakamoto and Vígh, 2007). Heat shock proteins (Hsps) are reported to be proteins that are usually expressed when responding to stress conditions (Guo et al., 2014). Especially, as an essential regulator of protein, the Hsp70 has the tendency to maintain internal cell stability, protecting the injured organisms by enhancing the stability of mRNA and translating preferentially under heat condition (McGarry and Lindquist, 1985). Previous studies showed that overexpression of Hsp70 gene was leading to increasing the tolerance of *Arabidopsis thaliana* (Shinya et al., 2014), *Capsicum annuum* (Guo et al., 2014; Usman et al., 2015), *Zea mays* (Ristic et al., 1991), and *O. sativa* (Wang et al., 2014) under heat and drought stress.

Although previous evidence indicated important roles of MeJA in regulating abiotic stress in plants, the function of MeJA in relation to heat tolerance remains unclear in perennial ryegrass. This study is aimed at investigating the effects of exogenous application of MeJA on osmotic adjustment, antioxidant defense, and the expression of JA-responsive genes after heat stress. The result would be useful to reveal possible MeJA-mediated mechanisms of heat tolerance and provides an option for alleviating heat damage in perennial ryegrass.

## Materials and Methods

### Plant Materials and Growth Conditions

Perennial ryegrass cultivar “Esquire” seeds were provided by DLF SEED A/S Company in China office. “Esquire” seeds were germinated in plastic pots (20 cm length, 15 cm width, and 10 cm height) filled with quartz sand and distilled water in a growth chamber at 20/15°C (day/night). The humidity and illumination were set to 70% relative and 750 μmol⋅m^–2^⋅s^–1^ PAR, respectively. After 7-day germination, seedlings were planted in Hoagland’s nutrient solution (Hope Bio-Technology Co., Ltd., Qingdao) for another 30 days. The pots’ positions were rearranged daily in order to reduce the impact of the environment.

### Treatments and Experimental Design

For exogenous MeJA concentration confirmation, a preliminary experiment was carried out with different MeJA concentrations (0, 20, 40, 60, 80, 100, 150, and 200 μmol⋅L^–1^) pretreatment on perennial ryegrass before heat stress. The results demonstrated that 100 μmol⋅L^–1^ MeJA had the most favorable effect on heat tolerance of perennial ryegrass under high-temperature stress, including relative water content (RWC), electrolyte leakage (EL), and total chlorophyll (Chl) content (Supplemental Figure 1). Accordingly, four treatments were designed for perennial ryegrass as follows: (1) Control check (labeled as CK), Hoagland’s nutrient solution with 20/15°C (day/night) temperature for 21 days; (2) Only MeJA pretreatment (labeled as CK + MeJA), plants were first pretreated with Hoagland’s nutrient solution containing 100 μmol/L MeJA with 20/15°C (day/night) for 7 days and then treated with Hoagland’s nutrient solution with 20/15°C (day/night) for 14 days; (3) High-temperature treatment (labeled as H), Hoagland’s nutrient solution with 20/15°C (day/night) temperature for 7 days, and then treated with 38/30°C (day/night) high-temperature for 14 days; (4) MeJA pretreatment and high-temperature treatment (labeled as H + MeJA), plants were first pretreated with Hoagland’s nutrient solution containing 100 μmol/L MeJA with 20/15°C (day/night) for 7 days and then treated with 38/30°C (day/night) high-temperature for 14 days. Each treatment had five biological replications. Samples were taken at 0 (labeled 0 day) and 14 (labeled 14 days) days after treatment with high temperature for all groups to analyze phenotypic and physiological variations. Samples from 0, 3, 6, 9, 12, and 24 h (labeled 0, 3, 6, 9, 12, and 24 h) after treatment with high temperature were used to analyze gene expression.

### Measurement

For RWC, the 0.2-g fresh leaves were taken as fresh weight (FW). Then, leaves were placed in distilled water at 4°C for 24 h and weighed to record saturated weight (SW). Subsequently, dried samples at 105°C for 30 min, followed by drying at 75°C for 48 h, and weighed to get dry weight (DW). RWC was calculated by the formula: RWC(%) = (FW−DW)/(SW−DW)×100%. The total Chl contents were extracted by incubating 0.1 g fresh leaves with a 10-ml solution of 80% acetone:95% methanol (1:1, V/V) in the dark until the leaves became colorless. The light absorption values of Chl a and Chl b were measured at 645 and 663 nm, respectively. The total Chl contents were calculated according to the following formula: Chl(a + b) (mg/g) = (20.2×*OD645* +  8.02×*OD663*)/(DW×1,000) (He and Gan, 2002). EL was determined by using a conductivity meter (Model 32, Yellow Springs Instrument Company). The method was clearly described in the study of Blum and Ebercon (1981).

For superoxide dismutase (SOD) activity, 1.5 ml of reaction solution [50 mM phosphate-buffered saline (PBS) containing 195 mM methionine, 60 μM riboflavin, and 1.125 mM nitro blue tetrazolium (NBT)] was mixed with 0.05 ml of enzyme extract, and then the reaction solution was placed under 800 μmol m^–2^ s^–1^ photosynthetically active radiation for 6 min. The absorbance was recorded at 560 nm (Giannopolitis and Ries, 1977). For peroxidase (POD) and catalase (CAT) activities, 0.05 ml of enzyme extract was added into 1.5 ml of reaction solution for POD determination or 1.5 ml of reaction solution for CAT assay (Chance and Maehly, 1955). For ascorbate peroxidase (APX) activity, the method was clearly described in the study of Nakano and Asada (1981). Changes in absorbance were monitored at 460, 240, or 290 nm every 10 s for 1 min for POD, CAT, or APX, respectively. To analyze malondialdehyde (MDA) content and antioxidant enzyme activities, 0.15 g of leaves was ground in 2 ml of 50 mM cold PBS (pH = 7.8). For MDA determination, 0.5 ml of supernatant was mixed with 1.0 ml of reaction solution and then incubated at 95°C for 15 min. The reaction solution was centrifuged at 8,000 *g* for 10 min, and the absorbance was measured at 532 and 600 nm (Dhindsa et al., 1981). The activity unit (U) of POD, CAT, and APX is defined as 0.01 changes within the first 1 min. Protein content was determined by using the method of Bradford (1976).

The plant JA ELISA Kit (MiBlo Inc., Shanghai, China) was used for the determination of JA and MeJA concentrations in plant tissue homogenates and other biological fluids. For both JA and MeJA content, measuring and setting the standard curve according to the kit procedures firstly, the linear regression equation of the calibration curve is calculated by using the concentration of the standard substance and the OD value. Here, 0.1 g of fresh leaves was ground with 0.01 m⋅ML^−1^ cold PBS (pH = 7.2–7.4) to get the supernatant after being centrifuged at 2000–3000 r⋅min^−1^ for 20 min at 4°C. Then, supernatant was treated following the manufacturer’s instructions, and the absorbance was measured at 450 nm. The OD value of the sample is substituted into the equation to calculate the sample concentration, and then multiplied by the dilution factor, which is the actual concentration of the sample.

### Identification of Related Genes in Perennial Ryegrass

In this study, four genes including *LpNAC022*, *LpNAC037*, *LpNAC045*, and *LpNAC054* from the NAC transcription factor families were selected, which play an important role in abiotic and biological stress response. Primer pairs for each gene were selected based on a previous published study (Nie et al., 2020). Another four genes from essential regulator of protein Hsp70 including *LpHsp70-009*, *LpHsp70-010*, *LpHsp70-015*, and *LpHsp70-020* were selected based on a previous study in our group, which is significant enhancing expression under heat stress condition. Furthermore, four genes in JA signal pathway including *LpLOX2*, *LpAOC*, *LpOPR3*, and *LpJMT* were identified, and the gene sequences were acquired from NCBI database of homologous genes including rice, wheat (*Triticum aestivum* L.), and corn. Alignment based on blast search with the conserved sequence and whole genome sequence of perennial ryegrass [downloaded from the Perennial Ryegrass Genome Sequencing Project^1^, (Byrne et al., 2015)] was conducted, and primer pairs for each gene were designed using the online tool Primer3^2^. All primer information was listed in Table 1.

**TABLE 1:** Primer information for all selected perennial ryegrass genes.

Gene	Forward primer (5′–3′)	Reverse primer (5′–3′)
LpAOC	CTACGAGGCCATCTACAGCA	AGGGGAAGACGATCTGGTTG
LpOPR3	AACCAAAGCAACCTTTCGCC	TTCAACCTGTTCCTGCGTCC
LpLOX2	GCACCATCGATGAGCGAAAC	ACCCCTGGCTCTGAAAATGG
LpJMT	CTTCGACCTCTCCCACCG	CTTCAACCTGTTCCTGCGTC
LpNAC022	ACGTTCCAAATAGGCAGTGG	TTCCCGTGCACCATGTATAA
LpNAC037	TCCTTTCGACGGAGCTTCTT	TCGTTATTAGCCTTGCACGC
LpNAC045	GCCGCCTCTACAACAAGAAG	TCGATGTCTGAGGAATCGTG
LpNAC054	TTGGGGAGAAGGAGTGGTTC	TTGCCGGAGTAGAAGACGAG
LpHSP70-009	GGTGTACGAGGGTGAGAGAG	CCTCCTGCACCATCTTCTCA
LpHSP70-010	CCTGCTGCTTGATGTCACTC	GGATGAGTACACCAGGCTGT
LpHSP70-015	GATCGTCGTCAAGCACAAGG	GCTGCGAGTCGTTGAAGTAG
LpHSP70-022	CATCATCAACGAGCCCACTG	ACCTCAAAGATGCCCTCCTC
eIF4A	AACTCAACTTGAAGTGTTG GAGTG	AGATCTGGTCCTGGAAAGA ATATG

### Total RNA Isolation and Quantitative Real-Time PCR Expression Analysis

Direct-zol^TM^ RNA MiniPrep Kit (Zymo Research Co.) was used for the extraction of total RNA according to the manufacturer’s instruction manual. Genomic DNA was eliminated using DNase I (G Zymo Research Co.). A NanoDrop ND-2000 spectrophotometer (Nano-Drop Technologies, Wilmington, DE, United States) was used to determine RNA concentration, purity, and integrity, followed by 1% agarose gel electrophoresis. iScript^TM^ cDNA (Bio-Rad Laboratories Inc.) was used for RNA reverse transcription following the manufacturer’s instructions. The quantitative RT-PCR technique was used to validate the expression of genes. A 10-μl mixture contained 5 μl of abm^®^ EvaGreen 2X qPCR Master Mix (Applied Biological Materials Inc., Canada), 1.5 μl of synthesized cDNA product, 0.3 μl of each primer, and 2.9 μl of ddH_2_O. The following qRT-PCR reaction protocol was used: an enzyme activation step at 95°C for 10 min with one cycle, denaturation at 95°C for 15 s, and anneal/extension at 60°C for 60 s, for a total of 35 cycles. To verify the specificity of each primer, Tm and melting-curve analysis was obtained (65 to 95°C with fluorescence measured every 0.5°C increment). Technical samples and biological samples were used for all qRT-PCRs (Nie et al., 2020). The relative gene expression level was analyzed according to the 2^−ΔΔCt^ method (Schmittgen and Livak, 2008), and eIF4A was set as the reference gene to standardize the expression data (Yan et al., 2014). SPSS 19.0 (IBM, Armonk, NY, United States) was employed to the analysis of variance (ANOVA) at the 0.05 probability level. Data were transformed to meet normality and homogeneity of variance. Fisher’s least significant difference (LSD) was used to determine differences between groups. Microsoft Excel 2007 (Microsoft, Redmond, WA, United States) was employed to generate the histograms used for data chart.

## Results

### Effects of Exogenous Methyl Jasmonic Acid on Leaf Relative Water Content, Electrolyte Leakage, and Chlorophyll Content of Perennial Ryegrass After Heat Stress

**FIGURE 1:**
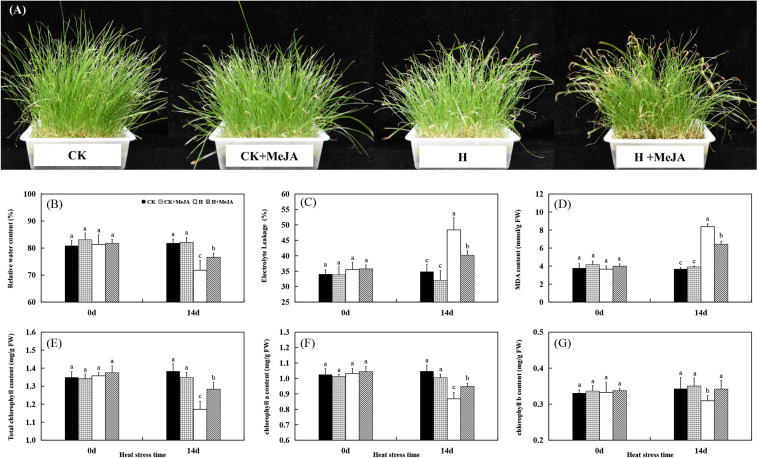
Effects of exogenous methyl jasmonic acid (MeJA) on phenotypic changes **(A)**, relative water content (RWC) **(B)**, electrolyte leakage (EL) **(C)**, malondialdehyde (MDA) content **(D)**, total chlorophyll content **(E)**, chlorophyll a content **(F)**, and chlorophyll b content **(G)** of perennial ryegrass after heat stress. Vertical bars indicate ± SE of the mean (*n* = 5). No common letter above bar indicates a significant difference by least significant difference (LSD) (*P* < 0.05). CK, control check; CK + MeJA, control check was pretreated with 100 μmol/L of MeJA; H, heat stress; H + MeJA, heat-stressed plants pretreated with 100 μmol/L of MeJA.

The morphological appearance of perennial ryegrass was shown in Figure 1A. Leaves turned yellow and wilting after 14 days of heat stress in H and H + MeJA group, but MeJA-pretreated plants in the H + MeJA group were greener than untreated plants. Exogenous MeJA pretreatment had no significant effects on all detected physiological indicators under well-temperature conditions (heat stress at 0 day). After 14 days of heat treatment, leaf RWC decreased while MeJA-pretreated plants exhibited significantly higher RWC than untreated plants (Figure 1B). Also, after 14 days of heat stress, the EL of MeJA-pretreated plants in the H + MeJA group showed 7.81% lower than untreated plants in the H group (Figure 1C). The 14 days of heat stress treatment increased MDA content, but exogenous MeJA application maintained significantly lower MDA content in the H + MeJA group than untreated plants in the H group in response to high temperature (Figure 1D).

For Chl content, the CK group maintained no significant difference with plant in well-temperature condition in the CK + MeJA group, while heat induced significant degradation of total Chl, Chl a, and Chl b contents. However, in the H + MeJA group, MeJA-pretreated plants exhibited observably higher total Chl, Chl a, and Chl b contents than those in the H group at 14 days of heat stress (Figures 1E–G). Exogenous MeJA application had a much significant effect on total Chl b content in leaves under heat condition in the H + MeJA group.

### Effects of Exogenous Methyl Jasmonic Acid on Endogenesis Jasmonic Acid and Methyl Jasmonic Acid of Perennial Ryegrass

**FIGURE 2:**
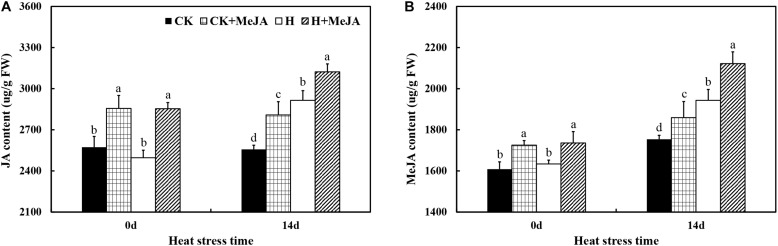
Effects of exogenous methyl jasmonic acid (MeJA) on JA content **(A)** and MeJA content **(B)** in leaves of perennial ryegrass after heat stress. Vertical bars indicate ± SE of the mean (*n* = 5). No common letter above bar indicates a significant difference by least significant difference (LSD) (*P* < 0.05) on a given day. CK, control check; CK + MeJA, control check was pretreated with 100 μmol/L of MeJA; H, heat stress; H + MeJA, heat-stressed plants pretreated with 100 μmol/L of MeJA.

After 7 days of exogenous MeJA application, it significantly increased the contents of endogenous JA and MeJA in leaves (Figure 2). After 14 days of high-temperature treatment, the content of endogenous JA in leaves increased about 13.99% in the H group compared with that in the CK group, while the highest endogenous content of JA was in the H + MeJA group after 14 days of heat stress. Also, the results showed that the endogenous JA content kept stable from 0 day to 14 days without heat stress treatment. For MeJA content, both heat stress and exogenous MeJA treatments could cause an accumulation of endogenous MeJA in leaves. Besides, compared with the MeJA content at 0 day, the endogenous MeJA content increased at 14 days in all groups, which was different with the JA biosyntheses and accumulation tendency.

### Effects of Exogenous Methyl Jasmonic Acid on Antioxidant Defense of Perennial Ryegrass After Heat Stress

**FIGURE 3:**
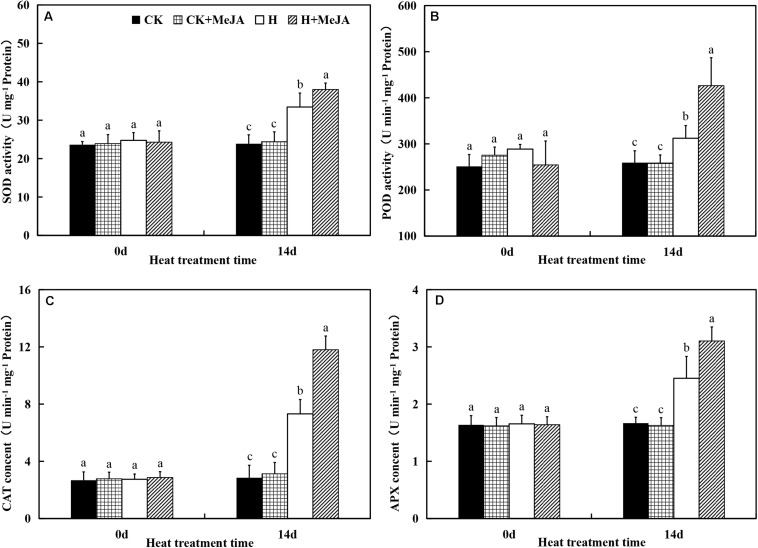
Effects of exogenous methyl jasmonic acid (MeJA) on (**A**) superoxide dismutase (SOD), (**B**) catalase (CAT), (**C**) peroxide (POD), and (**D**) ascorbate peroxidase (APX) activities in leaves of perennial ryegrass after heat stress. Vertical bars indicate ± SE of the mean (*n* = 5). No common letter above bar indicates a significant difference by least significant difference (LSD) (*P* < 0.05) on a given day. CK, control check; CK + MeJA, control check was pretreated with 100 μmol/L of MeJA; H, heat stress; H + MeJA, heat-stressed plants pretreated with 100 μmol/L of MeJA.

After 7 days of exogenous MeJA application, it did not have significant effects on SOD, CAT, POD, and APX activities. However, heat stress upregulated activities of SOD, CAT, POD, and APX in both H group and H + MeJA group after 14 days of high-temperature treatment (Figure 3). SOD activity significantly increased about 41.25% in the H group compared with the CK group after 14 days of heat stress, while plants in the H + MeJA group had significantly higher SOD activities than MeJA-untreated plants at 14 days after high-temperature treatment. Similar tendency was also found in POD, CAT, and APX activities. The results indicated that exogenous MeJA could enhance the antioxidant defense ability of plant leaves to resist the heat damage in perennial ryegrass.

### The Expression of Jasmonic Acid Biosynthesis Pathway-Related Genes in Perennial Ryegrass

**FIGURE 4:**
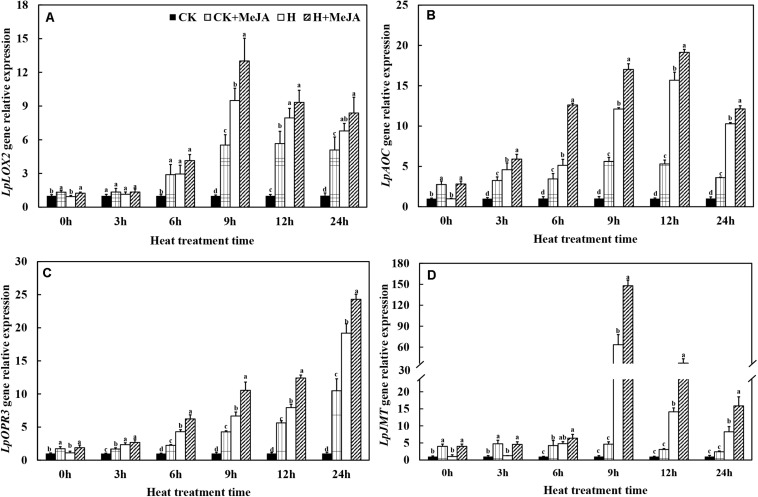
Effects of exogenous methyl jasmonic acid (MeJA) on relative expression of JA biosynthesis-related genes *LpLOX2* **(A)**, *LpAOC* **(B)**, *LpOPR3* **(C)**, and *LpJMT* **(D)** in leaves of perennial ryegrass after heat stress. Vertical bars indicate ± SE of the mean (*n* = 3). No common letter above bar indicates a significant difference by least significant difference (LSD) (*P* < 0.05) on a given day. CK, control check; CK + MeJA, control check was pretreated with 100 μmol/L of MeJA; H, heat stress; H + MeJA, heat-stressed plants pretreated with 100 μmol/L of MeJA.

After 7 days of exogenous MeJA pretreatment, the expressions of *LpLOX2*, *LpAOC*, *LpOPR3*, and *LpJMT* genes were improved at 0 h (Figure 4). Heat stress significantly upregulated transcript levels of these four genes in leaves of the H and H + MeJA groups, and the expression peak values of *LpLOX2*, *LpAOC*, and *LpJMT* were at 9 or 12 h (Figures 4A,B,D). A different expression profile was found in *LpOPR3*, which showed a continuous increased gene expression within 24 h when exposed to high-temperature condition in the H and H + MeJA groups (Figure 4C). Furthermore, the expression of *LpJMT* gene increased about 62 times in the H group than CK and about 32 times in the H + MeJA group than the CK + MeJA group after 9 h of high-temperature treatment, indicating a heat-induced gene involved in MeJA biosynthesis and accumulation.

### The Expression of NAC Transcription Factor and HSP-70 Protein Genes Responding to Heat and Methyl Jasmonic Acid Treatment

**FIGURE 5:**
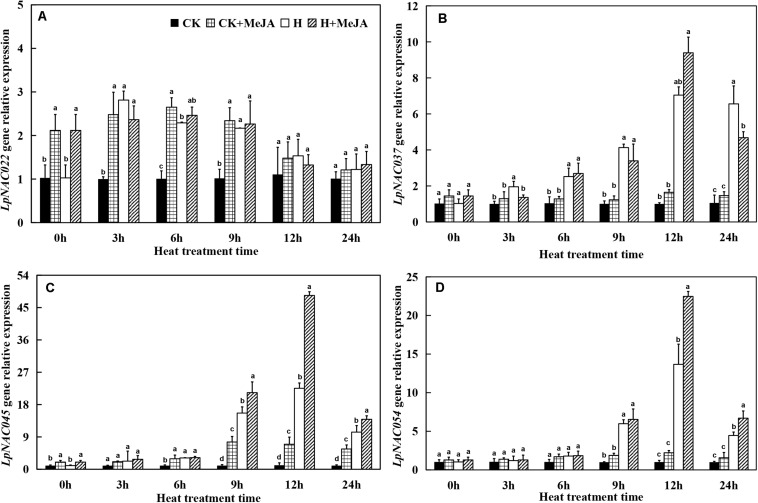
Effects of exogenous methyl jasmonic acid (MeJA) on relative expression of *LpNAC022* **(A)**, *LpNAC037* **(B)**, *LpNAC045* **(C)**, and *LpNAC054* **(D)** in leaves of perennial ryegrass after heat stress. Vertical bars indicate ± SE of the mean (*n* = 3). No common letter above bar indicates a significant difference by least significant difference (LSD) (*P* < 0.05) on a given day. CK, control check; CK + MeJA, control check was pretreated with 100 μmol/L of MeJA; H, heat stress; H + MeJA, heat-stressed plants pretreated with 100 μmol/L of MeJA.

At 0 h, the exogenous MeJA had no significant effect on gene expression levels of *LpNAC037* and *LpNAC054*, while the expression of *LpNAC022* and *LpNAC045* increased after 7 days of exogenous MeJA pretreatment (Figure 5). Heat stress upregulated the gene expression of *LpNAC037*, *LpNAC045*, and *LpNAC054* in leaves in the H and H + MeJA groups, and the peak value appeared at 12 h. However, the expression profile of *LpNAC022* gene was very different from the other three genes, and either exogenous MeJA or heat treatment could enhance the expression from 3 h. Overall, there was no obviously superimposed effect on MeJA- or heat-induced expression on *LpNAC022*, while *LpNAC037*, *LpNAC045*, and *LpNAC054* showed a significantly high expression in the H + MeJA group.

**FIGURE 6:**
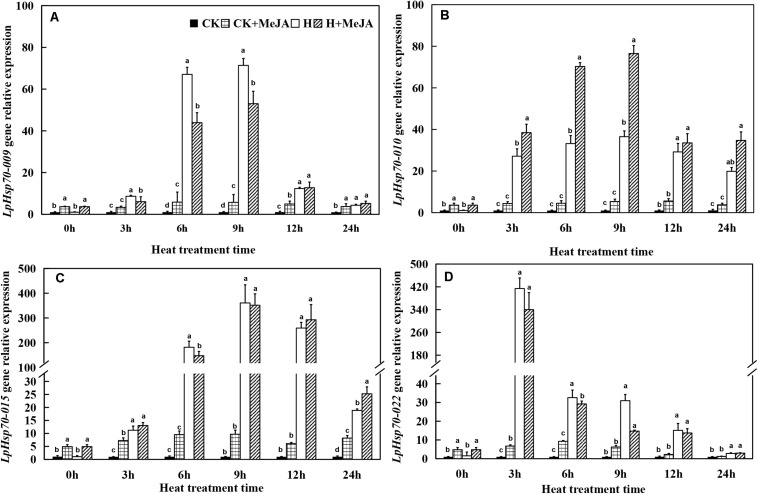
Effects of exogenous methyl jasmonic acid (MeJA) on relative expression of *LpHsp70-009* **(A)**, *LpHsp70-010* **(B)**, *LpHsp70-015* **(C)**, and *LpHsp70-022* **(D)** in leaves of perennial ryegrass after heat stress. Vertical bars indicate ± SE of the mean (*n* = 3). No common letter above bar indicates a significant difference by least significant difference (LSD) (*P* < 0.05) on a given day. CK, control check; CK + MeJA, control check was pretreated with 100 μmol/L of MeJA; H, heat stress; H + MeJA, heat-stressed plants pretreated with 100 μmol/L of MeJA.

For *LpHsp70* genes, although exogenous MeJA pretreatment could enhance the expression of these four genes at 0 h, it was obviously induced by high-temperature treatment when heat stress was applied (Figure 6). Among them, *LpHsp70-009*, *LpHsp70-010*, and *LpHsp70-015* genes in both the H and H + MeJA groups showed significantly high expression at 6 or 9 h, while *LpHsp70-022* gene showed a rapid response to high temperature and maximum expression values at 3 h.

## Discussion

Plants have evolved multiple acclimation mechanisms to survive environmental stresses such as heat stress. The accumulation of JAs is one of the mechanisms of plant resistance to abiotic stress (Wasternack and Hause, 2013). Previous studies have found that exogenous MeJA could increase Chl content and improve the tolerance of cowpea (*Vigna sinensis*) and *Brassica napus* (Ahmadi et al., 2018) under salinity stress (Sadeghipour, 2017). Besides, it could also enhance the ability for water stress resistance in cauliflower (*Brassica oleracea* L.) (Wu et al., 2012) and strawberry (*Fragaria x ananassa*) (Jordi and Leon, 2016). Furthermore, MeJA improved drought tolerance of soybean plants as a potential growth regulator (Mohamed and Latif, 2017). In this study, exogenous MeJA significantly increased the RWC of perennial ryegrass, while it decreased the EL under heat stress, which indicated a positive effect on perennial ryegrass to resistant high-temperature stress. At the same time, the exogenous MeJA contributed to maintaining the Chl content stability of perennial ryegrass leaves under heat stress, which may have a potential relationship with leave senescence (He et al., 2005).

In general, under appropriate growth environment, the content of reactive oxygen species (ROS) in plant cells is in a state of dynamic balance. Stress could cause a large amount of ROS accumulation, membrane lipid peroxidation, and finally lead to metabolic disorders in plants (Choudhury et al., 2013). The active oxygen scavenging system in plants is mainly composed of antioxidant enzymes (SOD, POD, APX, etc.) and antioxidant substances (Baxter et al., 2014). SOD is responsible for catalyzing disproportionation of O^2–^ into H_2_O_2_, and CAT, POD, and APX are mainly involved in H_2_O_2_ elimination (Cheruth et al., 2009). A large number of findings supported that exogenous JAs could enhance SOD, CAT, POD, and APX activity or gene expression, thereby alleviating oxidative damage and stabilizing cell membranes in plants under abiotic stress (Lehmann et al., 1995; Xin et al., 1997; Ghasempour et al., 1998). In this study, the activity of SOD, POD, CAT, and APX was further increased by exogenous MeJA treatment under heat condition and lower MDA content was accumulated, which was consistent with a previous study that the increased antioxidant enzyme activity favors the survival of plant to the heat stress and which induced the expression of related defense genes (Almeselmami et al., 2006).

Studies have shown that JA was a signaling molecule that regulates the expression of defense genes to various environmental stresses (Lehmann et al., 1995). Wasternack’s study (Wasternack, 2007) showed that the genes related to the JA signal pathway are all induced by JA substances, and the JA biosynthesis is regulated by positive feedback. Previous results showed that exogenous MeJA treatment significantly induced the upregulation of *LOX2* (AT3G45140) in the JA biosynthesis pathway of *A. thaliana* seedlings (Mao et al., 2017). The bulbs of *Gladiolus hybridus* were treated with different concentrations of exogenous MeJA, which could induce the upregulated expression of *GhAOS*, *GhAOC*, and 12-oxo-phytodienoic acid reductase 3 (*GhOPR3*) genes in the JA signal pathway, and the expression level rose with the MeJA concentration increasing (Lian et al., 2013). Also, JA is catalyzed by JA carboxyl methyl transferase (JMT) to form MeJA, and JMT could perceive and respond to local and systemic signals generated by external stimuli, including exogenous MeJA itself (Seo et al., 2001). In this study, the result showed that exogenous MeJA treatment could enhance the expression of the key genes in the JA pathway, including *LpLOX2*, *LpAOC*, *LpOPR3*, and *LpJMT.* Furthermore, they were all increased at different treatment points under heat condition, indicating that the key genes of the JA signal pathway have a positive response to the heat stress. Several reports also showed that endogenous JAs rapidly and massively accumulate under stress conditions in plants (Xin et al., 1997), and with the upregulated expression of key genes in the JA pathway, the content of endogenous JA and MeJA increased gradually (Song et al., 2014; Nie et al., 2020). These results indicated that heat stress and exogenous MeJA may both play a positive feedback regulatory role in the signal pathway of JAs, thus enhancing the concentration of endogenous JAs in leaves. When plants are under stress, JA treatment may make plants produce anti-stress reactions, including synthesizing proteins with special functions, inducing or activating related enzymes and producing secondary active substances, so as to resist the harm of adversity (Cai et al., 2006). Moreover, when plants are stimulated by the stress, they could induce the synthesis and accumulation of JAs directly or not.

NAC genes are also involved in plants responding to biotic and abiotic stress (Margaret and Thomas, 2001; Hu et al., 2006, 2008; Yoo et al., 2007). Previous study demonstrated that stress-responsive NAC (SNAC) proteins had important roles in the control of abiotic stress tolerance (Kazuo et al., 2012) and the expression level would increase when treated with methyl-jasmonate (Zhou et al., 2013). In this study, the results showed that the expression of *LpNAC037*, *LpNAC45*, and *LpNAC54* were significantly induced by heat stress, while the expression profile of *LpNAC022* gene was different with them. Besides, previous study had found that several stress-responsive transcription factors encoded by *SNAC* also played an important role in stress tolerance when treated with exogenous MeJA in *A. thaliana* (Ooka et al., 2003). Hsp70s are also essential in regulating plant growth, development, and stress response in almost all cells (Miernyk et al., 1992; Su and Li, 2008, 2010). Also, the overexpression of mitochondrial *HSP70* could inhibit the apoptosis induced by high temperature and oxidation in rice (Qi et al., 2011). In this study, the results showed that heat stress enhanced the expression of *LpHsp009*, *LpHsp70-010*, *LpHsp015*, and *LpHsp022* genes, while we could not see the significant difference with exogenous MeJA treatment. However, exogenous MeJA prompted expressions of *LpHsp010* gene, showing that this gene may have a relationship with MeJA under heat conditions.

## Data Availability Statement

The raw data supporting the conclusions of this article will be made available by the authors, without undue reservation.

## Author Contributions

GN and XZ conceived the project and designed the experiments. YS, YH, MT, JCa, and RW performed the experiments. YS and XD analyzed the data. YH, XD, and YS wrote the manuscript, and GN and XZ finalized at last. All authors discussed the results and reviewed the manuscript.

## Conflict of Interest

The authors declare that the research was conducted in the absence of any commercial or financial relationships that could be construed as a potential conflict of interest. The reviewer GY declared a past co-authorship with several of the authors, GN and XZ, to the handling editor.

## Footnotes

1
http://185.45.23.197:5080/ryegrassgenome

2 http://primer3.ut.ee/
