# Supplementary material for: Exogenous Methyl Jasmonate Improves Heat Tolerance of Perennial Ryegrass Through Alteration of Osmotic Adjustment, Antioxidant Defense, and Expression of Jasmonic Acid-Responsive Genes
Source: Front Plant Sci. 2021 May 7;12:664519. doi: 10.3389/fpls.2021.664519 (PMC8137847; doi:10.3389/fpls.2021.664519)
Supplement: Supplementary file 1 [file Presentation_1.PPTX]

## Slide 1
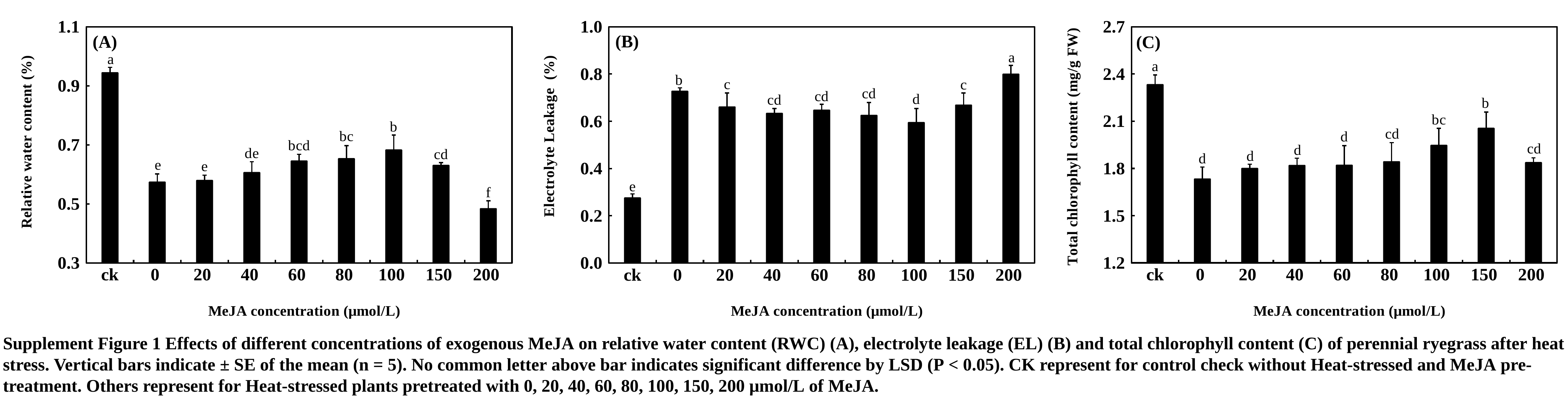

### Chart
| Category | |
|---|---|
| ck | 0.94408 |
| 0 | 0.57348 |
| 20 | 0.57924 |
| 40 | 0.60596 |
| 60 | 0.64502 |
| 80 | 0.65296 |
| 100 | 0.68244 |
| 150 | 0.63018 |
| 200 | 0.48354 |
### Chart
| Category | |
|---|---|
| ck | 0.27518 |
| 0 | 0.727 |
| 20 | 0.66042 |
| 40 | 0.6333 |
| 60 | 0.6467 |
| 80 | 0.62488 |
| 100 | 0.5944 |
| 150 | 0.66812 |
| 200 | 0.79928 |
### Chart
| Category | |
|---|---|
| ck | 2.3321 |
| 0 | 1.73276 |
| 20 | 1.79972 |
| 40 | 1.81866 |
| 60 | 1.82 |
| 80 | 1.84206 |
| 100 | 1.94646 |
| 150 | 2.05476 |
| 200 | 1.8369 |(B)
(A)
(C)
Supplement Figure 1 Effects of different concentrations of exogenous MeJA on relative water content (RWC) (A), electrolyte leakage (EL) (B) and total chlorophyll content (C) of perennial ryegrass after heat stress. Vertical bars indicate ± SE of the mean (n = 5). No common letter above bar indicates significant difference by LSD (P < 0.05). CK represent for control check without Heat-stressed and MeJA pre-treatment. Others represent for Heat-stressed plants pretreated with 0, 20, 40, 60, 80, 100, 150, 200 μmol/L of MeJA.
